# Peptidyl-Resin Substrates as a Tool in the Analysis of Caspase Activity

**DOI:** 10.3390/molecules27134107

**Published:** 2022-06-26

**Authors:** Remigiusz Bąchor

**Affiliations:** Faculty of Chemistry, University of Wroclaw, F. Joliot-Curie 14, 50-383 Wrocław, Poland; remigiusz.bachor@chem.uni.wroc.pl; Tel.: +48-71-375-7212; Fax: +48-71-328-2348

**Keywords:** caspases, substrate specificity, on-bead proteolysis, OBOC, fixed-charge tag, ESI-MS

## Abstract

Caspases, proteolytic enzymes belonging to the group of cysteine proteases, play a crucial role in apoptosis. Understanding their activity and substrate specificity is extremely important. Fluorescence-based approaches, including fluorogenic substrates, are generally used to confirm cleavage preferences. Here we present a new method of substrate specificity and activity analysis based on the application of fix-charge tagged peptides located on the resin. The proteolysis of peptide bond on the resin, occurring even with low efficiency, results in the formation of *N*-terminal fragments of model peptide containing ionization enhancers in the form of quaternary ammonium groups, allowing for ultrasensitive and reliable analysis by LC-MS/MS. The possibility of application of the proposed solution was tested through the analysis of substrate specificity and activity of caspase 3 or 7. The obtained results confirm the known substrate specificity of executioner caspases. Our solution also allowed us to observe that caspases can hydrolyze peptides shorter than those presented to date in the scientific literature.

## 1. Introduction

Proteolytic enzymes play key roles in essentially all signaling pathways, with infection and inflammation, apoptosis, blood clotting, and cell cycle control being classic examples. The misregulation of peptide and protein proteolysis may cause serious health disorders [1]. Therefore, the activity of some proteolytic enzymes may be treated as specific biomarkers for many diseases and a sensitive analytical method. The investigation of such activities requires specific substrates for analysis of enzyme kinetics. Several methods have been developed to define the optimal substrate specificity of proteases [2], and the results have been used to design substrates, inhibitors, or activity-based probes for several families of proteases [3]. Amongst them, the fluorescence-based solutions are used most widely, including the application of 7-amino-4-carbamoylmethylcoumarin (ACC). ACC was developed as a novel reagent that allows a fluorogenic molecule to be incorporated into a peptide scaffold as the C-terminal residue of peptides using standard solid phase synthetic methods [4]. Hydrolysis of the C-terminal amide bond results in release of free ACC and an ~900-fold increase in fluorescence intensity that is largely independent of pH, thus providing an efficient method to assay substrate specificity and enzyme activity. The ACC-containing combinatorial peptide libraries were successfully applied in the analysis of substrate specificity of different endopeptidases including caspases, neutrophil serine proteases, matrix metalloproteinases (MMPs) and cysteine cathepsins [5,6,7]. The FRET (Förster Resonance Energy Transfer) substrates and internally quenched fluorescent (IQF) peptide substrates constitute a convenient tool for examining the specificity of the largest group of proteases—endopeptidases [8]. The most commonly used IQF/FRET substrate pairs (donor/quencher) include Edans-Dabcyl, ABz-Tyr(NO2) [9], ABz-EDDNP [10], Trp-Dansyl [11], and 7-methoxy-coumarin-4-yl acetic acid-2,4-dinitrophenyl-lysine (MCA-Lys(DNP)) [12]. Recently, Drag and colleagues developed a new IQF pair composed of 7-amino-4-carbamoylmethylcoumarin (ACC) fluorophore and Lys(DNP), which was successfully applied in the determination of substrate specificity of caspases, elastase, legumain, MMP2 and MMP9, and trypsin endopeptidases [13].

Thornberry et al. [14], by using positional scanning synthetic peptide-based libraries, and Talanian and colleagues [15], using sets of individual peptide substrates, later refined by others, led to our current understanding of the inherent substrate preferences of caspases. It was presented that the peptide with DXXD/G (where D is Asp, X any amino acid, G is Gly and / denotes the cleavage site) sequence was found as a good candidate for caspase proteolysis. In addition the glutamic acid in the P1 position was found as an amino acid residue recognized by caspases in proteolysis; however, the hydrolysis was not as efficient as in the case of Asp [13,16]. 

All of the mentioned developments in the substrate specificity analysis of caspases described in-solution studies, while the commonly used One-Bead-One-Compound (OBOC) peptide combinatorial libraries include on-bead enzymatic digestion. The OBOC peptide libraries allow for obtaining and screening a wide range of compounds in a short time and are widely used in the investigation of new biologically active compounds [17,18]. Additionally, it was presented in the literature that the immobilized peptide-based conjugates may serve as tools for different compound capturing, analysis and transformation [19]. Previously, Bąchor et al. analyzed the possibility of the application of peptidyl-resin in the combinatorial chemistry using training combinatorial libraries for trypsin and chymotrypsin [19]. Currently, electrospray mass spectrometry (ESI-MS) is the method of choice for the identification of compounds on single beads. However, the necessity of analysis of a trace amount of peptide obtained from a single resin bead (about 10–15 mole) is insufficient for reliable sequence analysis. Previously, we reported on the application of the quaternary ammonium (QA) group as an ionization tag for ultrasensitive sequencing of peptides obtained from single resin beads of the OBOC peptide library by tandem mass spectrometry [20,21]. The described method is based on the application of both linear and bicyclic quaternary ammonium groups as ionization enhancers, located at a properly designed linker connecting the peptide with the resin. The applicability of the proposed strategy in combinatorial chemistry was confirmed using a training library of α-chymotrypsin substrates. 

Introduction of a QA group used as a fixed charge tag increases the ionization efficiency of compounds during ESI-MS analysis and significantly lowers the detection limit up to the attomolar level [22,23]. However, up to now, the analysis of on-bead peptide proteolysis and cleavage sites have not been investigated by MS due to the necessity of analysis of trace amounts of compound obtained from a single resin bead, which may be insufficient for reliable sequence analysis. The identification of peptide fragments released to the supernatant after peptide bond hydrolysis occurring on the resin is usually characterized by low yield [24]. Therefore, due to the small range of liberated peptides and their low ionization efficiency during the ESI-MS experiment, their reliable identification may be limited. The aim of this work was to develop an ultrasensitive method of substrate specificity analysis of caspase-3 and -7 using the LC-ESI-MS/MS technique and resin-bound peptides modified at the *N*-terminus by ionization tags.

## 2. Results and Discussion

The main goal of this work was to develop a new method of substrate specificity and activity analysis based on the application of fix-charge tagged peptides located on the resin and identification of proteolysis products in solution using the LC-MS/MS method. Due to the low yield of proteolysis occurring on the solid phase, we observed a small range of released compounds with generally low ionization efficiency during ESI-MS analysis. Therefore, to overcome this problem, we decided to apply the ionization enhancer in the form of the 4-aza-1-azoniabicyclo[2.2.2]octylammonium acetyl group, a bicyclic quaternary ammonium group at the N-terminus of the caspase substrate. The on-resin peptide bond cleavage by caspase, occurring even with low yield, results in the formation of a peptide fragment containing the quaternary ammonium group, able to be identified in supernatant by the ESI-MS technique.

**Figure 1:**
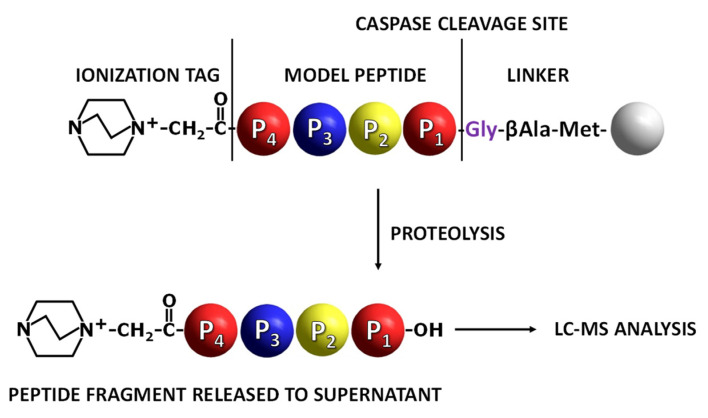
Application of fixed-charge tag peptidyl-resin in the analysis of caspase activity. Grey ball indicates TentaGel resin bead [19].

To prove the concept, the model peptide sequences containing known substrates for caspase 3 or 7 derivatized by fixed-charge tag (QA) at the *N*-terminus were synthesized on the TentaGel HL-NH_2_ resin. Peptide sequence was separated from the resin by the linker containing Met (cleavage site in the reaction with cyanogen bromide [19]), βAla (spacer) and Gly (P1′ residue) (Figure 1). After the synthesis of the ionization tag, side chain deprotection was performed [19], and the peptidyl-resin was used for caspase activity analysis. All ESI-MS/MS spectra of obtained model peptides are presented in the Supplementary Materials (Figures S1–S6).

**Figure 2:**
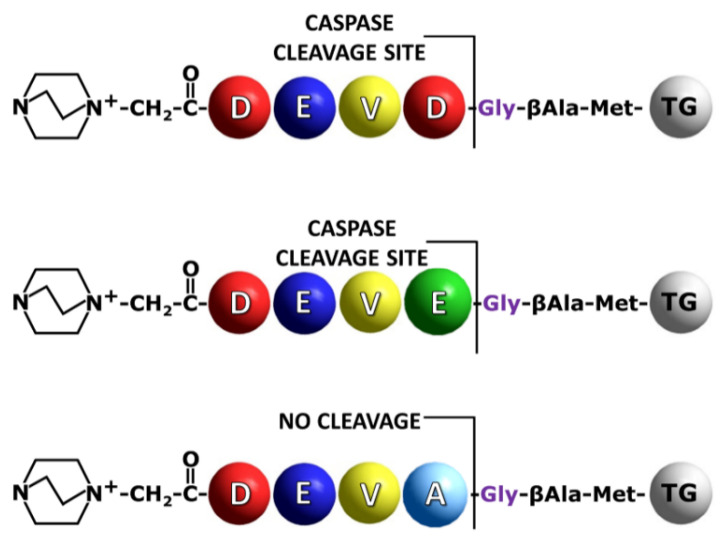
Schematic presentation of the synthesized model sequences containing QA group for the on-bead analysis of peptide bond hydrolysis by caspases. TG indicates TentaGel resin bead.

In our investigation, the DEVD/G sequence was used as a model, allowing us to observe high proteolytic activity of caspase 3 or 7 due to its high recognition by the enzymes used [14] (Figure 2). Additionally, the DEVE/G sequence was synthesized to determine the possibility of its cleavage on the resin and sensitive identification of the fixed charge tag fragment in solution by the LC-MS/MS technique. The DEVE/G was able to be hydrolyzed, however, poorly, as mentioned before [13,16,19].

**Figure 3:**
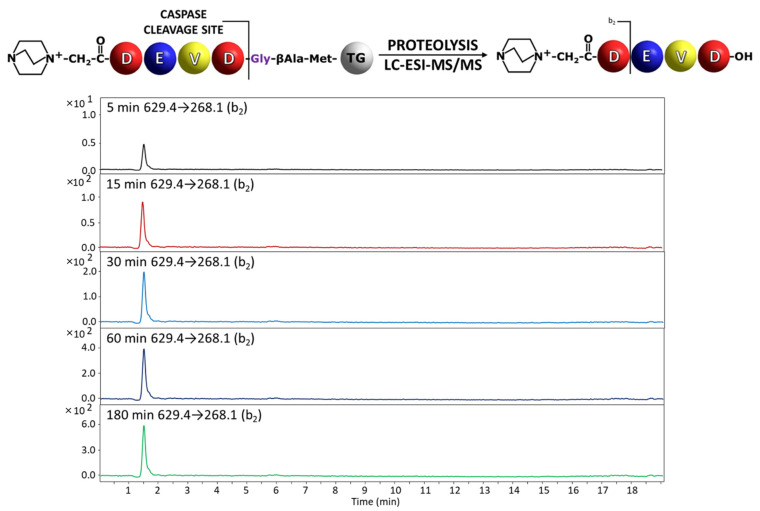
Chromatograms for QA-DEVD-OH peptide investigated in supernatant after incubation with caspase 3. Analyzed transition 629.4→268.1.

**Figure 4:**
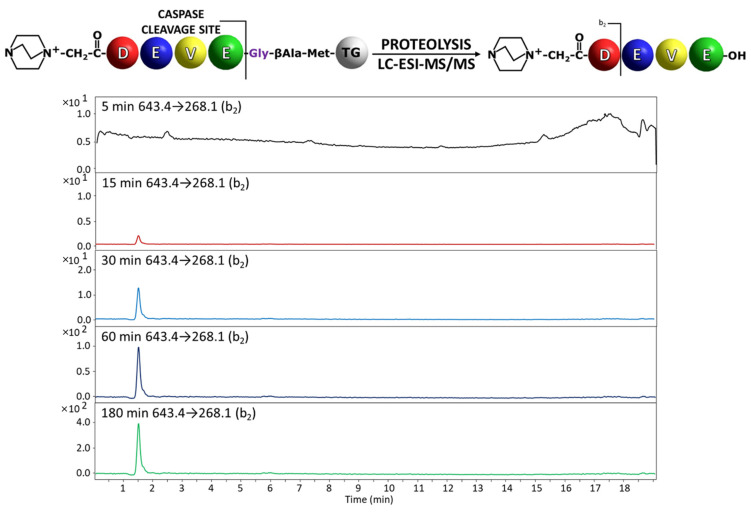
Chromatograms for QA-DEVE-OH peptide investigated in supernatant after incubation with caspase 3. Analyzed transition 629.4→268.1.

The synthesized model peptides were derivatized at the *N*-terminus by the fixed charge tag in the form of 4-aza-1-azoniabicyclo[2.2.2]octylammonium acetyl group using a previously presented method [25]. After the side chain deprotection the peptidyl-resin was prepared for the reaction with an enzyme. The portions of 0.5 mg of the obtained peptidyl-resins, modified by ionization enhancer, after swelling in buffer solution containing 20 mM Pipes (2,2′-(Piperazine-1,4-diyl)di(ethane-1-sulfonic acid)), 0.1 M NaCl, 1 mM EDTA, 10 mM DTT, and 10% (w/v) sucrose at pH 7.2 to 7.4, were incubated separately with a caspases 3 or 7 for 5, 15, 30, 60 and 180 min. Then, the formic acid (20 µL) was added to terminate the enzymatic reaction. The obtained supernatant was decanted, lyophilized and analyzed by mass spectrometry. In order to increase the sensitivity of detection, the multiple reaction monitoring (MRM) mode was applied. All the model compounds were synthesized on Wang resin, purified by HPLC and analyzed by ESI-MS/MS (Supplementary Materials, Figures S7–S9). The following transitions were investigated: for QA-DEVD-OH: 629.4→268.1 (b_2_), 629.4→240.1 (a_2_), 643.4→222.1 (a_2_-H_2_O), 643.4→196.1 (a_2_-CO_2_); QA-DEVE-OH: 643.4→268.1 (b_2_), 643.4→240.1 (a_2_), 643.4→196.1 (a_2_-CO_2_), 643.4→153.0 (b_1_); QA-DEVA-OH: 585.3→268.1 (b_2_), 585.3→240.1 (a_2_), 585.3→196.1 (a_2_-CO_2_), 585.3→153.0 (b_1_). For each of the obtained compounds, the most intense signal on the MRM chromatograms corresponds to the b_2_ ion. Therefore, this ion was selected to monitor the progress of the enzymatic hydrolysis on a solid support by MRM [19]. The high intensity of b_2_ ion results from the formation of stable succinimide anhydride as a consequence of amide bond dissociation after aspartic acid residue according to the charge remote fragmentation mechanism [26]. The obtained chromatograms for the most intensive transition are presented in Figure 3 and Figure 4.

In the analysis of the peptidyl-resin containing QA-DEVD/G sequence, the presence of the released fragment with QA-DEVD sequence was identified in the supernatant even after 5 min of enzymatic reaction (Figure 3). The hydrolysis after Glu residue in the P1 position in the case of peptidyl-resin with the QA-DEVE/G sequence was also determined in the supernatant, after 15 min of incubation with enzyme (Figure 4). Thus, the proposed method confirmed the previous observations regarding the preferences of the applied caspases in the recognition and hydrolysis of substrates containing Asp and Glu residues in the P1 position [13,19]. Even the hydrolysis in the presence of Glu residue is limited by the enzyme specificity and also by the solid support; the proposed method, due to its high sensitivity related to the presence of ionization tag, allows for the analysis of enzyme activity [19].

The peptidyl-resin containing the QA-DEVA/G sequence was not hydrolyzed, as expected, and therefore the signal corresponding to the released peptide fragment was not observed. The obtained results confirmed the known specificity and activity of used caspases.

**Figure 5:**
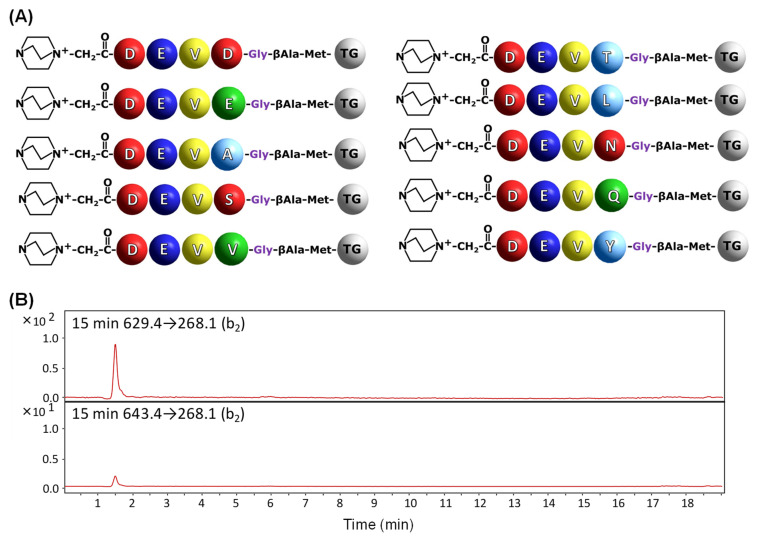
Schematic presentation of model training library for caspase 3 (**A**) and MRM chromatograms (**B**) for QA-DEVD-OH (629.4→268.1) and QA-DEVE-OH (643.4→268.1) peptides investigated in supernatant after 15 min of enzymatic digestion.

In order to confirm the applicability of the developed method in the analysis of caspase substrate specificity and activity towards substrates attached to the resin, the model training peptide library containing 10 components was synthesized (Figure 5A). Two of the obtained components should be hydrolyzed by caspase 3. The sequences were designed so that all resulting peptides differ in molecular weight, which facilitated their identification by MS. The obtained mass spectrum for library components is presented in the Supplementary Materials (Figure S10). The obtained library containing QA-modified compounds was treated by caspase 3 for 15 min, and the collected supernatant was analyzed by the LC-MS-MRM method. The obtained chromatograms are presented in the Figure 5B.

The obtained results (Figure 5B) clearly confirm the on-bead digestion of two model peptides with QA-DEVD and QA-DEVE sequences by caspase 3 after 15 min of enzymatic reaction. Low intensity of the signal corresponding to the MRM transition 643.4→268.1 is a consequence of limited proteolysis after glutamic acid residue in comparison to that observed for QA-DEVDG-peptidyl resin. To give clear evidence of the influence of applied fixed-charge tags on the ionization efficiency and possibility of analysis of trace amounts of sample in the supernatant after enzymatic digestion of peptidyl-resin a model, N-terminally acetylated peptidyl-resin with the Ac-DEVD-G-βA-M-TG (TG—TentaGel) was synthesized and subjected to enzymatic digestion in the presence of caspase 3 or caspase 7 in separate experiments according to the presented procedure. Collected supernatants were then analyzed by LC-MS; however, the signals corresponding to the released fragments were not identified (Figure S11). The obtained data clearly present the usefulness of the fixed-charge tag in the ultrasensitive analysis of small amounts of compounds characterized also by low ionizability by mass spectrometry.

In 1997, Thornberry and colleagues [14] described the optimal tetrapeptide recognition motif with the DXXD/G sequence for caspases. All the peptidic substrates for caspases consist of tetrapeptides as efficiently processed, even those containing non-proteinogenic amino acids [6,27,28]. To test the possibility of cleavage of the shorter peptides by caspases, we decided to synthesize shorter analogues of DEVD/G sequences containing an ionization tag at the *N*-terminus (Table 1). A total of 5 mg of the prepared peptidyl-resin was incubated with caspase 3 or 7 in separate experiments for 15, 30 and 60 min. Then, after the enzymatic cleavage was terminated, the supernatant was separated from the resin beads and analyzed by the LC-MS-MRM method.

**Figure 6:**
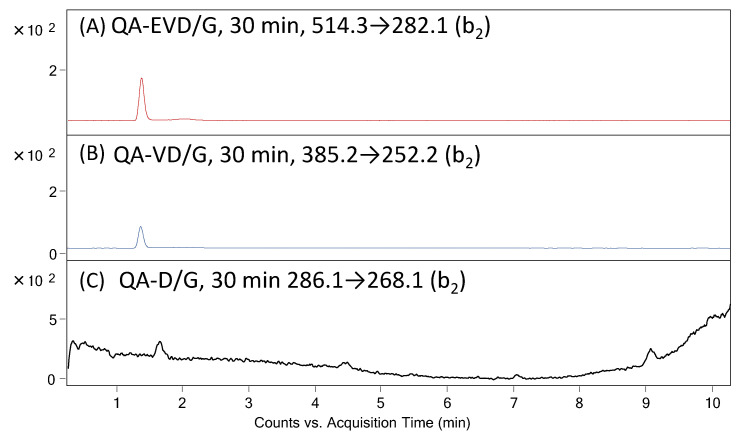
MRM chromatograms for (**A**) QA-EVD-OH (514.3→282.1), (**B**) QA-VE-OH (385.2→252.2) and (**C**) QA-D/G peptides investigated in supernatant after 30 min of enzymatic digestion on solid support.

It was found that in the case of peptides with the QA-EVD/G and QA-VD/G sequences, the cleavage of the D/G bond was observed after 30 min of enzymatic reaction, as confirmed by the presented LC-MS-MRM data (Figure 6). The hydrolysis of peptides with the QA-D/G sequence was not observed, probably due to the presence of only one residue at the P1 and P1′ position.

S.J Martin in 2014 presented [29] that caspases can cleave proteins containing specific tetrapeptide sequences with an almost absolute preference for aspartic acid at the scissile bond (P1 site). According to Thornbery and colleagues [14], the preferred tetrapeptide sequence cleaved by caspase-3 and caspase-7 is DXXD (where ‘X’ represents any amino acid). Each caspase has its own preference for a particular tetrapeptide sequence, but substitution with chemically similar amino acids is usually tolerated at several positions within the recognition motif. The consistent feature of each cleavage site is an Asp (D) residue in the P1 position and a small amino acid in the P2 position. Later, it was found that glutamic acid can also be located in the P1 position in peptides recognized by the caspases [30,31]. Moreover, identification of the preferred cleavage site and peptide sequence does not explain the mechanism of protein cleavage by caspases [28]. The use of shorter motifs than tetrapeptides for substrate specificity or/and activity analysis has never been described. The performed investigation led to the conclusion that caspase 3 and 7 can recognize and hydrolyze even the peptide with the QA-VE sequence located on the resin. Additionally, the obtained data suggest the positive charge located within the ionization tag at the *N*-terminus of the model peptide does not affect the enzymatic activity.

Previously, we found that the hydrogen atoms located at the alpha carbon atom within the 4-aza-1-azoniabicyclo[2.2.2]octylammonium acetyl group are able to be exchanged to deuterons under basic conditions [32]. The introduced deuterons do not undergo back exchange under acidic and neutral conditions, and the isotopologues present co-elution. This allowed us to prepare isotopically labeled standards of different compounds for their quantitative analysis by LC-MS [33,34,35]. Therefore, the presented tool for analysis of caspase activity and specificity may also be used in a quantitative way after preparation of appropriate deuterated QA-modified peptide sequences.

## 3. Materials and Methods

### 3.1. Reagents

All solvents and reagents were used as supplied. Fmoc amino acid derivatives were purchased from Novabiochem (Billerica, MA, USA). 2-(1H-7-Azabenzotriazol-1-yl)-1,1,3,3-tetramethyluro noium hexafluorphosphate (HATU), formic acid, trifluoroacetic acid (TFA) and N,N’-diisopropylcarbodiimide (DIC) were obtained from IrisBiotech. TentaGel^®^ HL-NH_2_ resin (0.56 mmol/g, 110 μm particle size) was purchased from RappPolymere (Tuebingen, Germany). Cyanogen bromide (3M solution in dichloromethane), 1,4-diazabicyclo[2.2.2]octane (DABCO), iodoacetic acid, triisopropylsilane (TIS), Pipes (2,2′-(Piperazine-1,4-diyl)di(ethane-1-sulfonic acid)), ethylenediaminetetraacetic acid (EDTA), DL-dithiothreitol (DTT), caspases 3 and 7 and solvents for peptide synthesis (*N,N*-dimethylformamide (DMF), dichloromethane (DCM), and (N-ethyldiisopropylamine (DIEA)) were obtained from Sigma Aldrich (St. Louis, MO, USA).

### 3.2. Peptide Synthesis and QA Group Formation

Synthesis of model peptides was performed manually in polypropylene syringe reactors (Intavis AG) equipped with polyethylene filters, according to a standard Fmoc (9-fluorenylmethoxycarbonyl) solid phase synthesis procedure [36]. Briefly, to the syringe containing 50 mg of the resin in DMF, 4 equivalents of Fmoc-amino acid, 4 equivalents of coupling reagent (HATU) and 8 equivalents of DIEA dissolved in DMF were added (Supplementary Materials, Figure S12). Fmoc protecting group was removed under basic conditions (25% solution of piperidine in DMF). Coupling efficiency was controlled by the Kaiser test. After the peptide synthesis, the N-terminus was iodoacetylated using the mixture of iodoacetic acid (26 mg, 140 μmol) and DIC (17.7 mg, 140 μmol), dissolved in DMF (0.5 mL) and added to the peptidyl resin (50 mg, 28 μmol); the reaction was repeated 3 times for 30 min at room temperature. Then, the peptidyl resin was washed with DMF (7 × 1 min) and the 1,4-diazabicyclo[2.2.2]octane (DABCO) (31.4 mg, 280 μmol) dissolved in DMF (1 mL) was added and mixed overnight (Supplementary Materials, Figure S12). Then, the resin was washed with DMF (7 × 1 min), DMF/DCM (1:1; *v*:*v*, 1 min), DCM (3 × 1 min), DCM/MeOH (1:1; *v*:*v*, 1 min) and MeOH (3 × 1 min) and dried in vacuo. Side chain protecting groups were cleaved by the mixture of TFA/TIS/H_2_O (95:2.5:2.5; *v*:*v*:*v*) for 2 h.

### 3.3. Peptide Cleavage

The dry peptidyl resin (0.5 mg) was treated with 0.25 M BrCN in 70% formic acid (200 μL) for 18 h, according to the method described by Franz et al. [37]. The obtained product was dissolved in a mixture (100 μL) of water, acetonitrile and formic acid (50:50:0.1; *v*:*v*:*v*) and analyzed directly by ESI-MS.

### 3.4. Enzymatic Digestion

The peptidyl resin (0.5 mg) was washed with water (3 × 1 min) and buffer (3 × 1 min) containing 20 mM Pipes, 0.1 M NaCl, 1 mM EDTA, 10 mM DTT, and 10% (w/v) sucrose at pH 7.2 to 7.4. Before the reaction with peptidyl resin, the solutions of caspases 3 or 7 (concentration 100 μM) were preincubated for 15 min at 37 °C. Enzymatic digestion was performed for 5, 15, 30, 60 and 180 min in 37 °C. The peptidyl resin to enzyme ratio was 1000:1 (w/w). The reaction was terminated by the addition of formic acid (10 μL). The supernatant was separated manually, and the resin was washed with water (3 × 1 min), acetonitrile (3 × 1 min) and methanol (3 × 1 min) and dried in vacuo.

### 3.5. Mass Spectrometry

All ESI-MS and LC-MS/MRM experiments were performed on the LCMS-8050 Shimadzu apparatus (Shimadzu, Kyoto, Japan), equipped with the UHPLC Nexera X2 system. The Aeris Peptide XB-C18 column (50 mm × 2.1 mm) with a 3.6 μm bead diameter and equilibrated at 24 °C, was used. The LC system was operated with a mobile phase consisting of solvent A: 0.1% formic acid in H_2_O and solvent B: 0.1% formic acid in MeCN. The gradient conditions (B %) were from 5 to 80% B within 40 min. The flow rate was 0.1 mL/min, and the injection volume 5 μL. The instrument parameters were as follows: scan range: 100–1000 *m*/*z*; drying gas: nitrogen; flow rate: 1.5 L/min; temperature: 300 °C; potential between the spray needle and the orifice: 4.2 kV. The LC system was operated with the following mobile phase: A: 0.1% formic acid in H_2_O and solvent B: 0.1% formic acid in MeCN; the gradient conditions (B %) were from 5 to 60% B within 14 min. The flow rate was 0.1 mL/min, and the injection volume 0.1 μL.

The MRM method was optimized automatically, and the following transitions were chosen: for QA-DEVD-OH: 629.4→268.1 (b_2_), 629.4→240.1 (a_2_), 643.4→222.1 (a_2_-H_2_O), 643.4→196.1 (a_2_-CO_2_); QA-DEVE-OH: 643.4→268.1 (b_2_), 643.4→240.1 (a_2_), 629.4→196.1 (a_2_-CO_2_), 629.4→153.0 (b_1_); QA-DEVA-OH: 585.3→268.1 (b_2_), 585.3→240.1 (a_2_), 585.3→196.1 (a_2_-CO_2_), 585.3→153.0 (b_1_); QA-EVD-OH: 514.3→282.1 (b_2_), QA-VE-OH: 385.2→252.2 (b_2_) QA-D-OH 286.1→268.1 (b_1_).

## 4. Conclusions

A method of caspase activity and specificity by mass spectrometry using the peptidyl-resin approach and derivatization strategy to increase the sensitivity of detection was developed. The proposed solution is based on the idea of fixed-charge tag derivatization of the N-terminal part of the resin-bound peptide, which after the enzymatic digestion, will be presented in the solution, ready for LC-MS analysis. The peptidyl-resins containing known substrates for caspase 3 or caspase 7 were synthesized. The peptide bond cleavage after aspartic acid was confirmed. Additionally, the presence of quaternary ammonium tags allowed the identification of peptide fragments released to the solution after Glu-Gly bond cleavage due to the enhanced ionizability. The method allowed us to confirm the known substrate specificity of caspases. The obtained results clearly confirmed the applicability of the proposed method, as the known substrate specificity of the applied caspases was confirmed. Additionally, the proposed approach allowed us to confirm that caspases are able to hydrolyze shorter fragments than tetrapeptides. The presented solution may allow for the ultrasensitive analysis of new caspase substrates and caspase activity. The proposed method could revolutionize the combinatorial search for new substrates of proteolytic enzymes. The proposed approach will overcome the problems connected to insufficient amounts of peptide released from a single resin bead and may be widely used for discovering new substrates of proteolytic enzymes. Such substrates may serve as new tools for analysis of biomarkers of several diseases and disorders.

## Figures and Tables

**Table 1:** Mass spectrometry data of the investigated peptides with the QA-EVD/G, QA-VD/G and QA-D/G sequences.

Nr	Peptide Sequence	M^+^	MRM Transition
1	QA-EVD/G	514.3	282.1
2	QA-VD/G	385.2	252.2
3	QA-D/G	286.1	268.1

## Data Availability

The data presented in this study are available in the Supplementary Materials.

## Supplementary Materials

The following supporting information can be downloaded at: https://www.mdpi.com/article/10.3390/molecules27134107/s1, Figure S1: ESI-MS/MS spectrum of DABCO+CH2CO-Asp-Glu-Val-Asp-Gly-β-Ala-Hsl peptide conjugate. Parent ion m/z 840.10, collision energy 50 eV. Hsl–homoserine lactone; Figure S2: ESI-MS/MS spectrum of DABCO + CH2CO-Asp-Glu-Val-Asp-Gly-β-Ala-Hsl conjugate. Parent ion m/z 420.50, collision energy 15 eV. Hsl–homoserine lactone; Figure S3: ESI-MS/MS spectrum of DABCO+CH2CO-Asp-Glu-Val-Glu-Gly-β-Ala-Hsl conjugate. Parent ion m/z 854.10, collision energy 60 eV. Hsl–homoserine lactone; Figure S4: ESI-MS/MS spectrum of DABCO+CH2CO-Asp-Glu-Val-Glu-Gly-β-Ala-Hsl conjugate. Parent ion m/z 427.60, collision energy 15 eV. Hsl–homoserine lactone; Figure S5: ESI-MS/MS spectrum of DABCO+CH2CO-Asp-Glu-Val-Ala-Gly-β-Ala-Hsl conjugate. Parent ion m/z 796.20, collision energy 50 eV. Hsl–homoserine lactone; Figure S6: ESI-MS/MS spectrum of DABCO+CH2CO-Asp-Glu-Val-Ala-Gly-β-Ala-Hsl conjugate. Parent ion m/z 398.60, collision energy 15 eV. Hsl–homoserine lactone; Figure S7: ESI-MS/MS spectrum of DABCO+CH2CO-Asp-Glu-Val-Asp-OH conjugate. Parent ion m/z 629.28, collision energy 40 eV; Figure S8: ESI-MS/MS spectrum of DABCO+CH2CO-Asp-Glu-Val-Glu-OH conjugate. Parent ion m/z 643.28, collision energy 40 eV; Figure S9: ESI-MS/MS spectrum of DABCO+CH2CO-Asp-Glu-Val-Ala-OH conjugate. Parent ion m/z 585.29, collision energy 40 eV; Figure S10: ESI-MS spectrum of model combinatorial library; Figure S11: Chromatograms for QA-DEVE-OH peptide investigated in supernatant after incubation with caspase 3. Analyzed transition 519.2→158.1; Figure S12: Schematic presentation of solid-phase peptide synthesis and derivatization with quaternary ammonium group.
