# Supplementary material for: Peptidyl-Resin Substrates as a Tool in the Analysis of Caspase Activity
Source: Molecules. 2022 Jun 26;27(13):4107. doi: 10.3390/molecules27134107 (PMC9268085; doi:10.3390/molecules27134107)
Supplement: Supplementary file 1 [file molecules-27-04107-s001.zip › molecules-1751240-supplementary.pdf]

Supplementary data

Peptidyl-resin substrates as a tool in the analysis of caspase activity

Remigiusz Bąchor

Faculty of Chemistry, University of Wroclaw, F. Joliot-Curie 14, 50-383 Wrocław, Poland

Corresponding Author

* Remigiusz Bąchor, Faculty of Chemistry, University of Wrocław, F. Joliot-Curie 14, 50-383 Wrocław, Poland, Tel.: +48-71-375-7212; Fax: +48-71-328-2348

E mail: remigiusz.bachor@chem.uni.wroc.pl

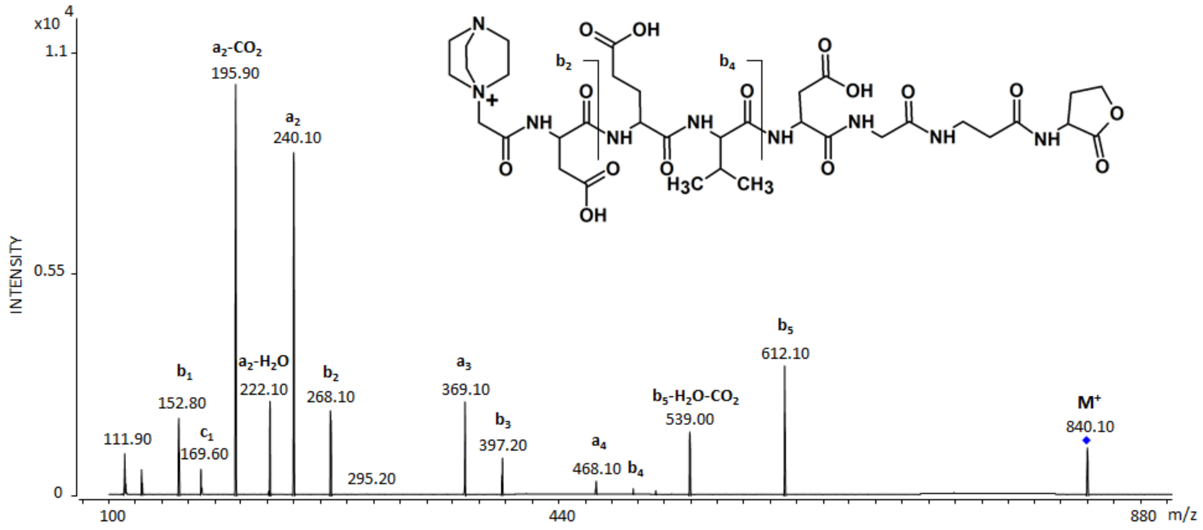

Figure S1. ESI-MS/MS spectrum of DABCO^{+}CH$_2$CO-Asp-Glu-Val-Asp-Gly-β-Ala-Hsl peptide conjugate. Parent ion m/z 840.10, collision energy 50 eV. Hsl–homoserine lactone.

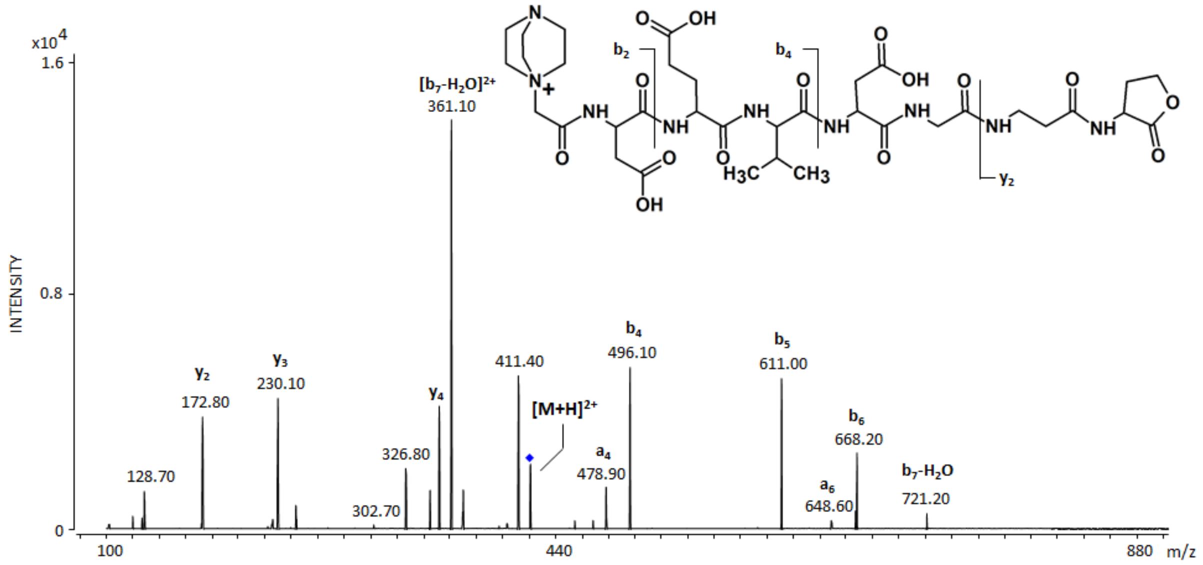

Figure S2. ESI-MS/MS spectrum of DABCO^{+}CH$_2$CO-Asp-Glu-Val-Asp-Gly-β-Ala-Hsl conjugate. Parent ion m/z 420.50, collision energy 15 eV. Hsl–homoserine lactone.

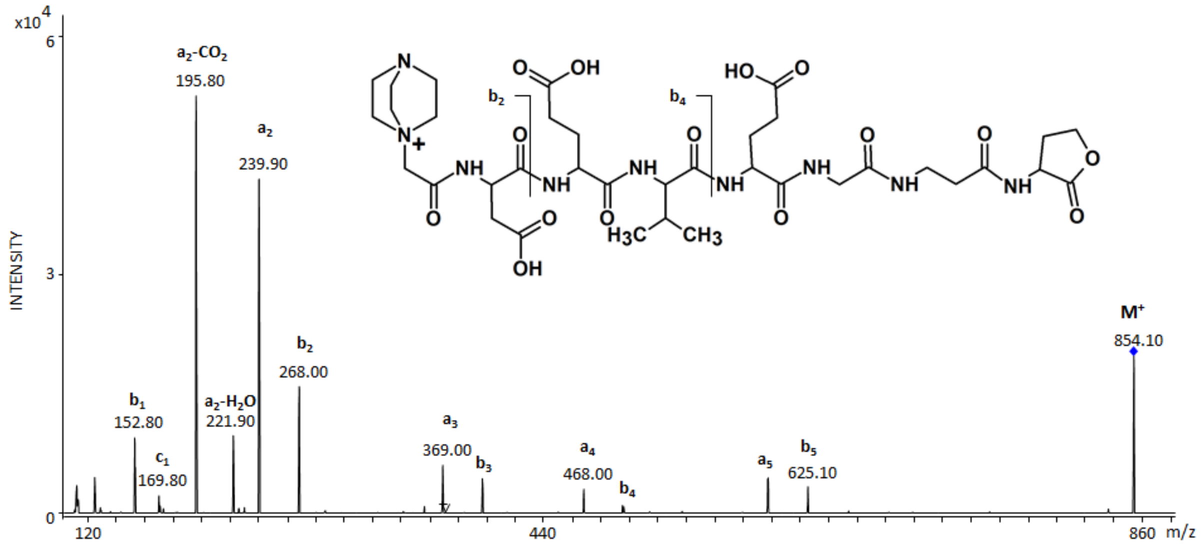

Figure S3. ESI-MS/MS spectrum of DABCO$^+$CH$_2$CO-Asp-Glu-Val-Glu-Gly-β-Ala-Hsl conjugate. Parent ion m/z 854.10, collision energy 60 eV. Hsl–homoserine lactone.

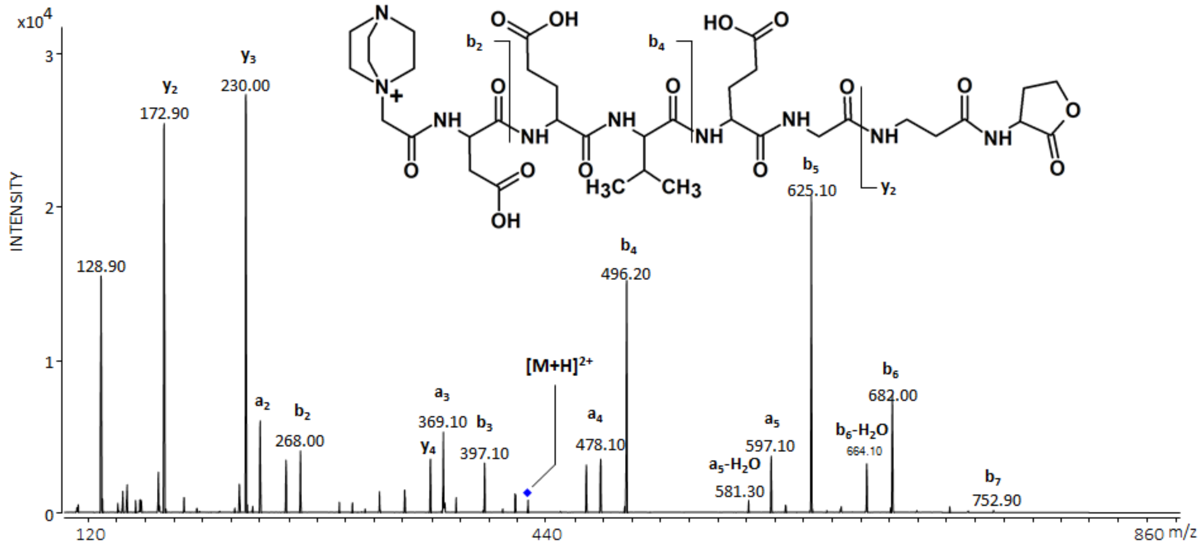

Figure S4. ESI-MS/MS spectrum of DABCO$^+$CH$_2$CO-Asp-Glu-Val-Glu-Gly-β-Ala-Hsl conjugate. Parent ion m/z 427.60, collision energy 15 eV. Hsl–homoserine lactone.

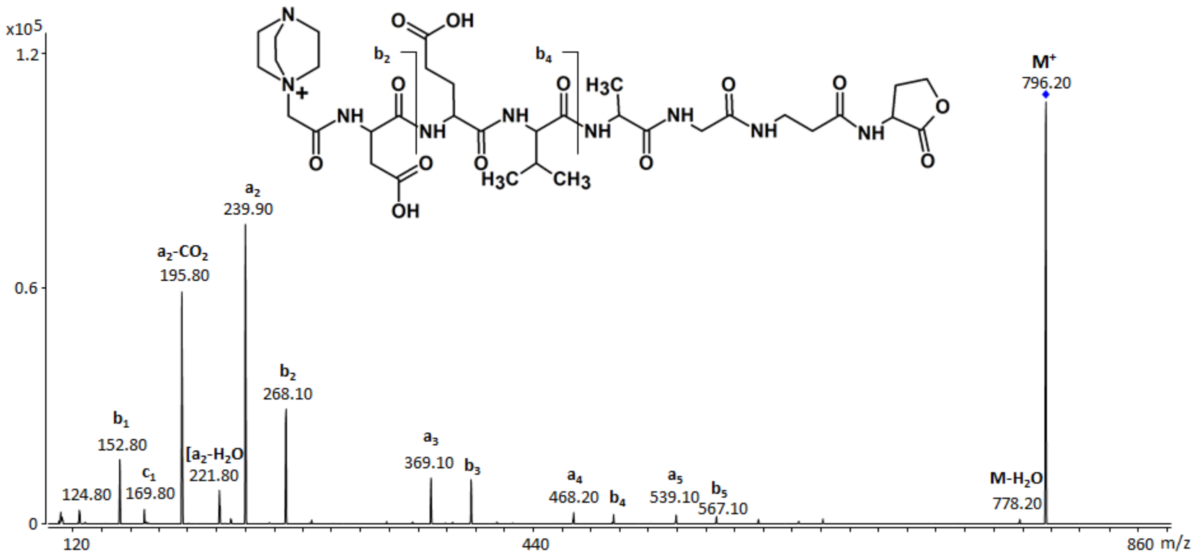

Figure S5. ESI-MS/MS spectrum of DABCO$^+$CH$_2$CO-Asp-Glu-Val-Ala-Gly-β-Ala-Hsl conjugate. Parent ion m/z 796.20, collision energy 50 eV. Hsl–homoserine lactone.

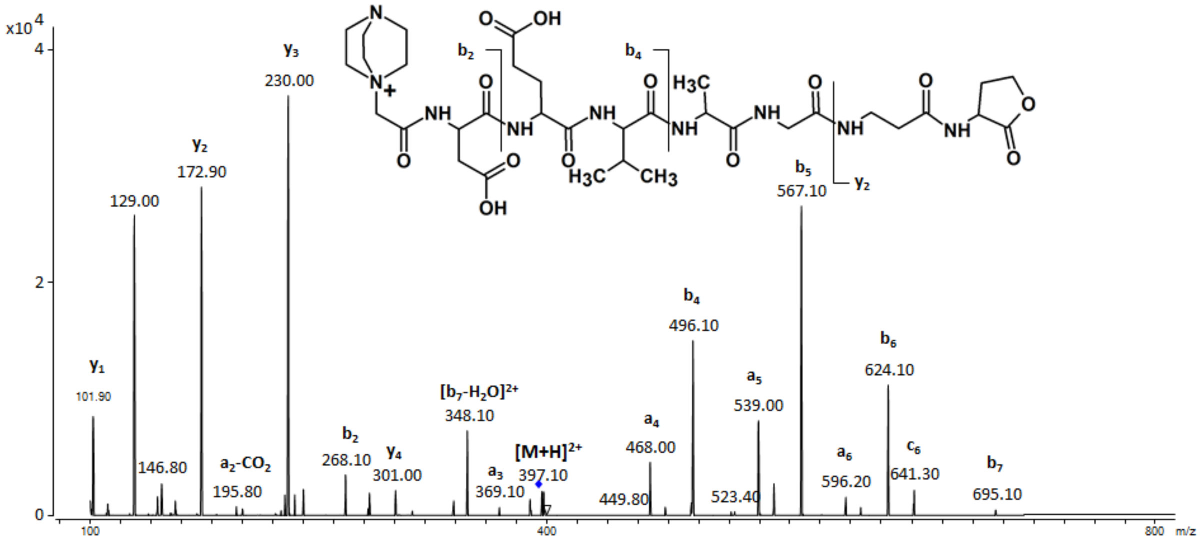

Figure S6. ESI-MS/MS spectrum of DABCO$^+$CH$_2$CO-Asp-Glu-Val-Ala-Gly-β-Ala-Hsl conjugate. Parent ion m/z 398.60, collision energy 15 eV. Hsl–homoserine lactone.

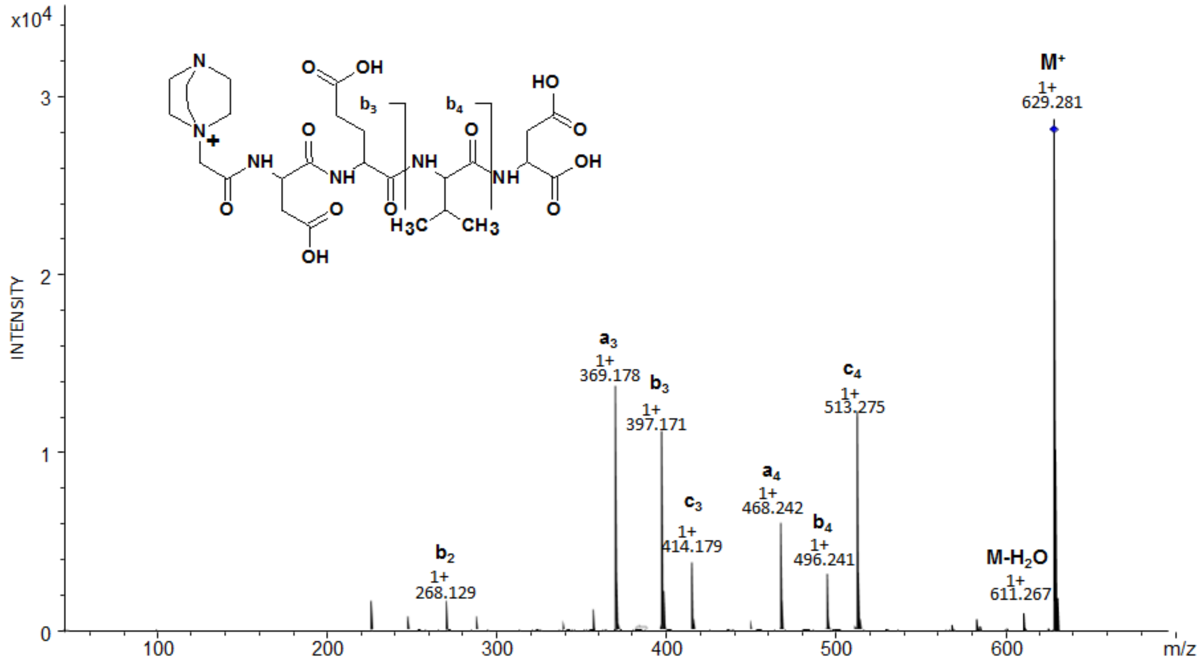

Figure S7. ESI-MS/MS spectrum of DABCO$^+$CH$_2$CO-Asp-Glu-Val-Asp-OH conjugate. Parent ion m/z 629.28, collision energy 40 eV.

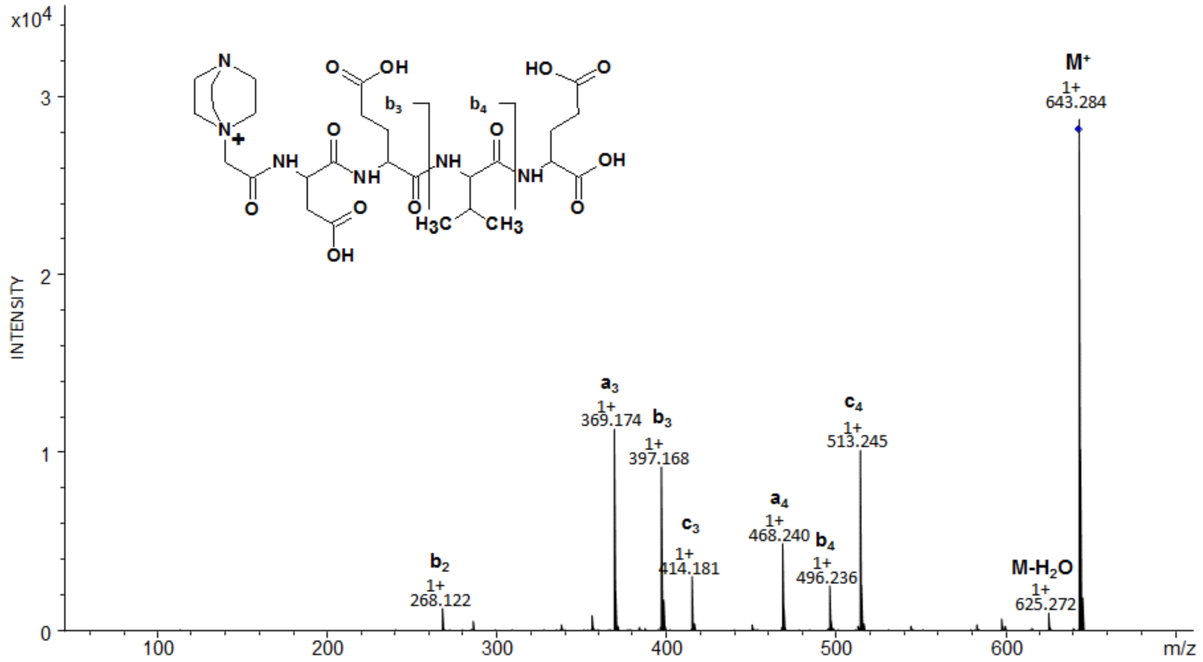

Figure S8. ESI-MS/MS spectrum of DABCO$^+$CH$_2$CO-Asp-Glu-Val-Glu-OH conjugate. Parent ion m/z 643.28, collision energy 40 eV.

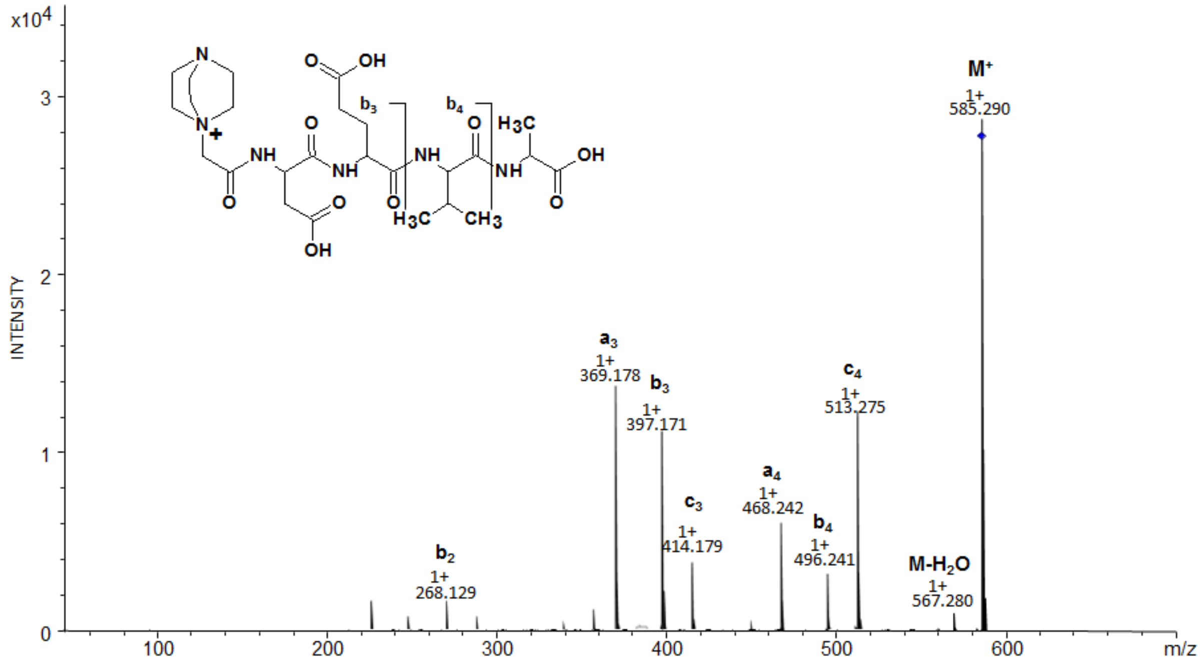

Figure S9. ESI-MS/MS spectrum of DABCO^{+}CH$_2$CO-Asp-Glu-Val-Ala-OH conjugate. Parent ion m/z 585.29, collision energy 40 eV.

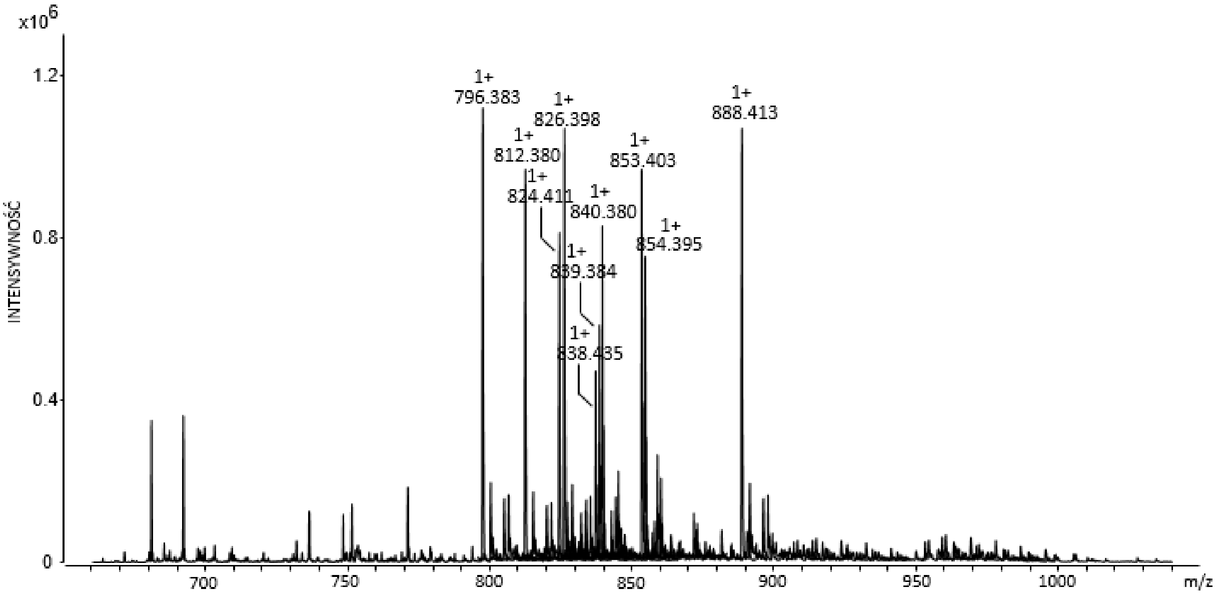

Figure S10. ESI-MS spectrum of model combinatorial library.

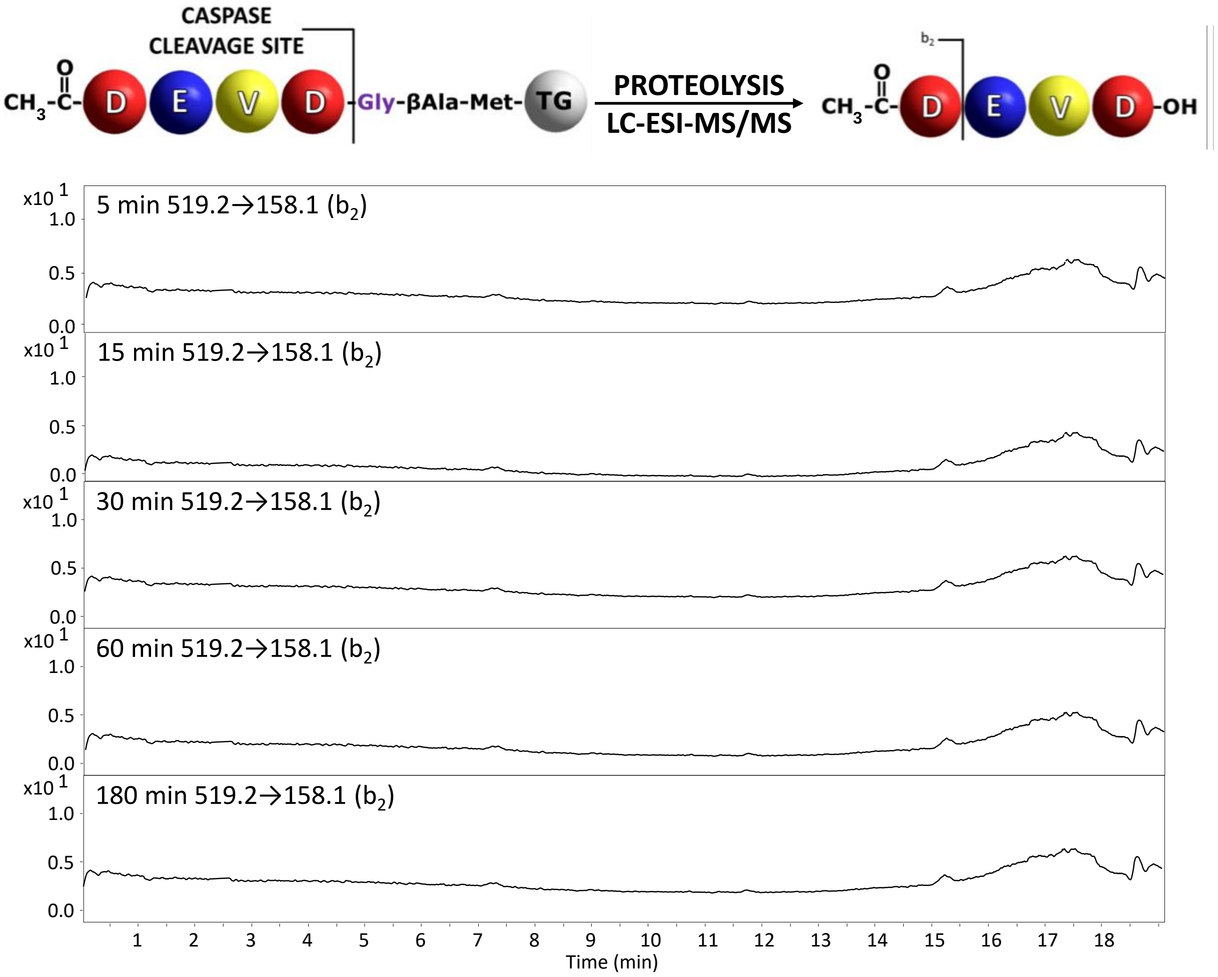

Figure S11. Chromatograms for QA-DEVE-OH peptide investigated in supernatant after incubation with caspase 3. Analyzed transition 519.2→158.1.

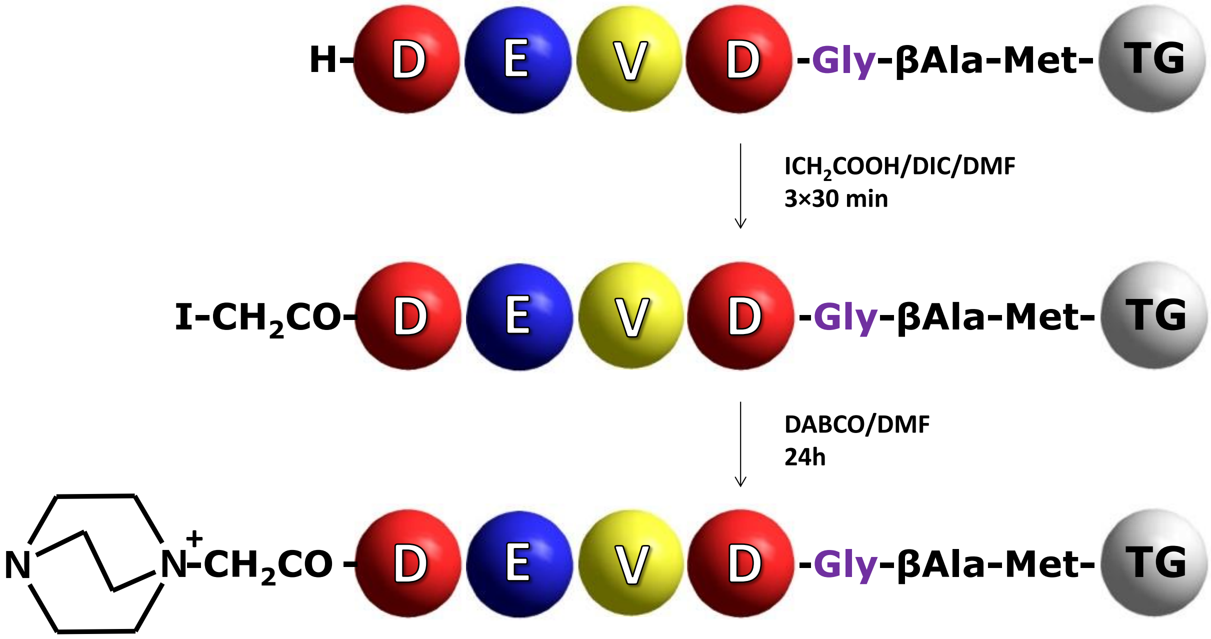

Figure S12. Schematic presentation of solid-phase peptide synthesis and derivatization with quaternary ammonium group.
